# Distal Stent Graft-Induced New Entry after Total Arch Replacement with Frozen Elephant Trunk for Aortic Dissection

**DOI:** 10.3400/avd.oa.21-00105

**Published:** 2021-12-25

**Authors:** Yoshikatsu Nomura, Shuto Tonoki, Motoharu Kawashima, Jun Fujisue, Gaku Uchino, Shunsuke Miyahara, Hiroshi Tanaka, Tasuku Honda, Nobuhiko Mukohara, Hirohisa Murakami

**Affiliations:** 1 Department of Cardiovascular Surgery, Hyogo Brain and Heart Center at Himeji, Himeji, Hyogo, Japan

**Keywords:** aortic dissection, frozen elephant trunk, distal stent graft-induced new entry

## Abstract

**Objectives:** Distal stent graft-induced new entry (dSINE), defined as a new tear caused by a stent graft, has been increasingly observed following total arch replacement using frozen elephant trunk (FET) for aortic dissection. We aimed to investigate the incidence and treatment of dSINE after the use of FET.

**Methods:** This retrospective study evaluated 70 patients who underwent total arch replacement using FET for aortic dissection between August 2014 and March 2020. They were followed up for at least 6 months postoperatively. Between-group comparisons were performed between those who did and did not develop dSINE. The risk factors for the development of dSINE and the treatment of dSINE were analyzed.

**Results:** dSINE occurred postoperatively in nine patients (12.9%) with a median time frame of 17.7±11.7 months. The incidence of dSINE did not differ significantly according to classification, phase of dissection, or oversizing. All patients in the dSINE group developed enlargement of the false lumen. dSINE closure was successfully achieved without complications via thoracic endovascular aortic repair (TEVAR) in all patients.

**Conclusion:** No independent factors predicting the development of dSINE were noted in this study. Additional TEVAR for dSINE provides good results and achieves false lumen thrombosis in the thoracic aorta, with no complications.

## Introduction

In 1996, Kato et al. first reported the insertion of a home-made stent graft (SG) in a descending thoracic aortic aneurysm or the true lumen in the case of an aortic dissection.^1)^ In 2003, Karck et al. named this method frozen elephant trunk (FET).^2)^ Thereafter, the FET method has been widely used as an effective treatment for aortic dissection.^3–5)^ FET is mainly indicated for acute type A aortic dissection, especially when the intimal tear is located in the aortic arch or proximal descending aorta; distal malperfusion; and young patients. Other indications include chronic aortic dissection, acute type B dissection contraindicated for endovascular treatment, and chronic type B aortic dissection contraindicated for left thoracotomy or requiring the median sternal approach. However, the risk of secondary aortic reinterventions following FET remains significant. Distal stent graft-induced new entry (dSINE) is a particular problem in the follow-up period, occurring in 15.8%–18% of cases.^6,7)^

Frozenix (Japan Lifeline, Tokyo, Japan), which was a commercially available Japan-made FET prosthesis, was released in 2014, and more than 12,000 prostheses have been implanted.^8)^ This study aimed to evaluate the incidence and risk factors of dSINE in patients treated using the Frozenix device and report the results of thoracic endovascular aortic repair (TEVAR) for dSINE.

## Patients and Methods

### Study design and patients

This retrospective study evaluated 88 patients who underwent total arch replacement (TAR) with FET for aortic dissection at our center between August 2014 and March 2020. Among them, 70 patients who could be followed up for more than 6 months were included.

Frozenix was used in all patients. The dissection phase at the time of surgery was acute in 44 patients, subacute in 6, and chronic in 20. Residual dissection following ascending aortic replacement was defined as Stanford type A aortic dissection. The phasing of aortic dissection was based on the Japanese 2020 Guidelines on the Diagnosis and Treatment of Aortic Aneurysm and Aortic Dissection: acute within 2 weeks, subacute within 2 weeks–3 months, and chronic after 3 months of onset.^9)^ TAR with FET was performed for chronic type B aortic dissection when left thoracotomy was contraindicated or when the median sternal approach was required.

This study was approved by our institutional review board (IR No. R3-20). The need for informed consent was waived due to the retrospective nature of the study.

### Surgical procedure and FET size selection

**Figure 1:**
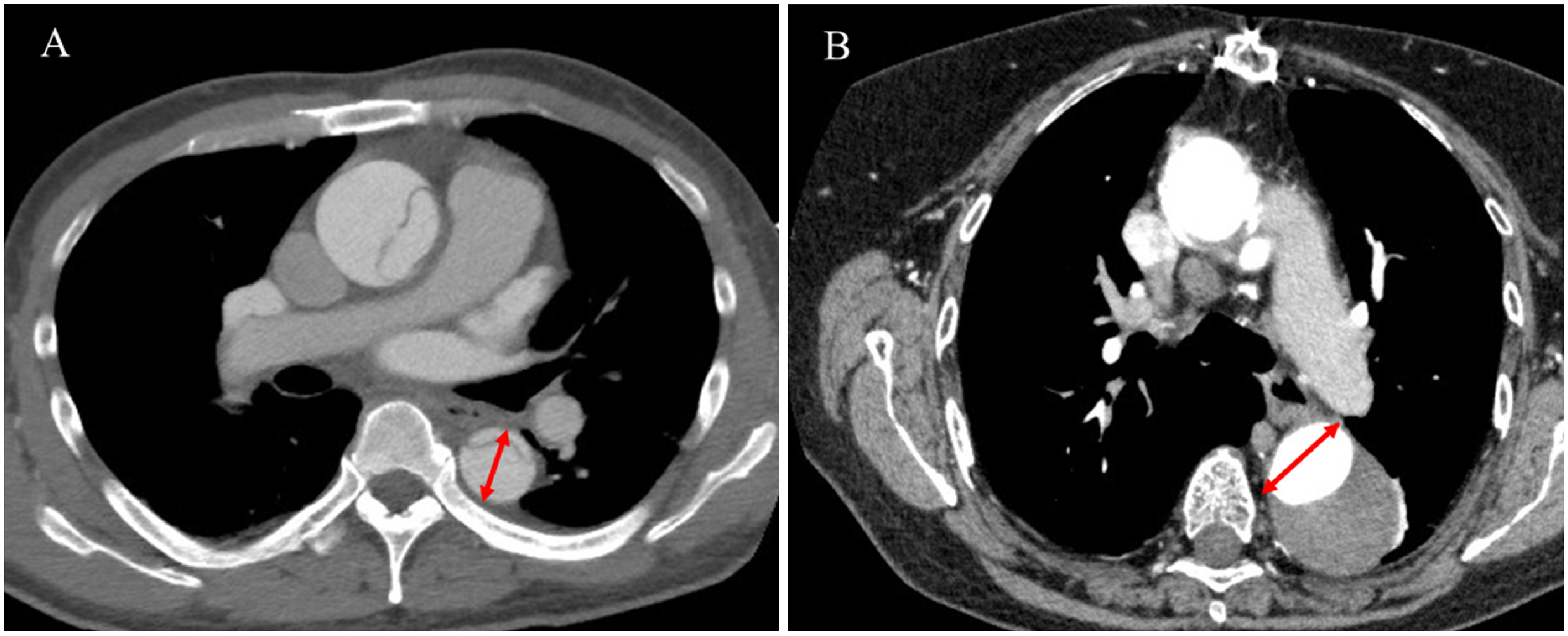
Fig. 1 Preoperative computed tomography images. (**A**) Acute aortic dissection: The whole aortic diameter of the planned frozen elephant trunk (FET) implantation site is measured (double-headed arrow). (**B**) Chronic dissection (postoperative residual dissection): The true lumen long-axis diameter of the FET site was measured (double-headed arrow).

TAR with the FET was performed through median sternotomy. The arterial cannulation site for the cardiopulmonary bypass was the femoral artery and/or axillary artery for acute type A dissection and the ascending aorta for type B dissection. At a rectal temperature of 28°C, after circulatory arrest, the aorta was incised, and selective antegrade cerebral perfusion was initiated. The distal anastomosis site of the TAR was set at the distal end of the left subclavian artery. To avoid spinal cord ischemia (SCI), the implantation position was set proximally so as not to exceed the aortic valve, that is, the Th8 level. Given that the aorta at the distal end of the left subclavian artery was dissected and inserted into the true lumen, the FET length was set to 6, 9, or 12 cm. Ultimately, the length was selected based on the distal anastomosis, location of the entry, and surgeon’s preference. The size of the FET selected was below 80% of the whole aortic diameter of the planned implantation site in acute dissection and 100%–110% of the true lumen diameter in chronic dissection (**Fig. 1**). The FET was inserted into the true lumen, the nonstented portion was removed, a Felt strip was wrapped around the circumference of the aorta, and the aorta and FET were fixed with several stitches. The distal side was anastomosed with a four-branched graft. The proximal and cervical branches were then reconstructed.

### Imaging analysis

Contrast-enhanced computed tomography (CT) was performed postoperatively before discharge. Plane CT was performed at 3 months, 6 months, 1 year, and annually thereafter. If the false lumen (FL) was expanded or FET migration into the FL was suspected, contrast-enhanced CT was employed to confirm the presence of dSINE.

**Figure 2:**
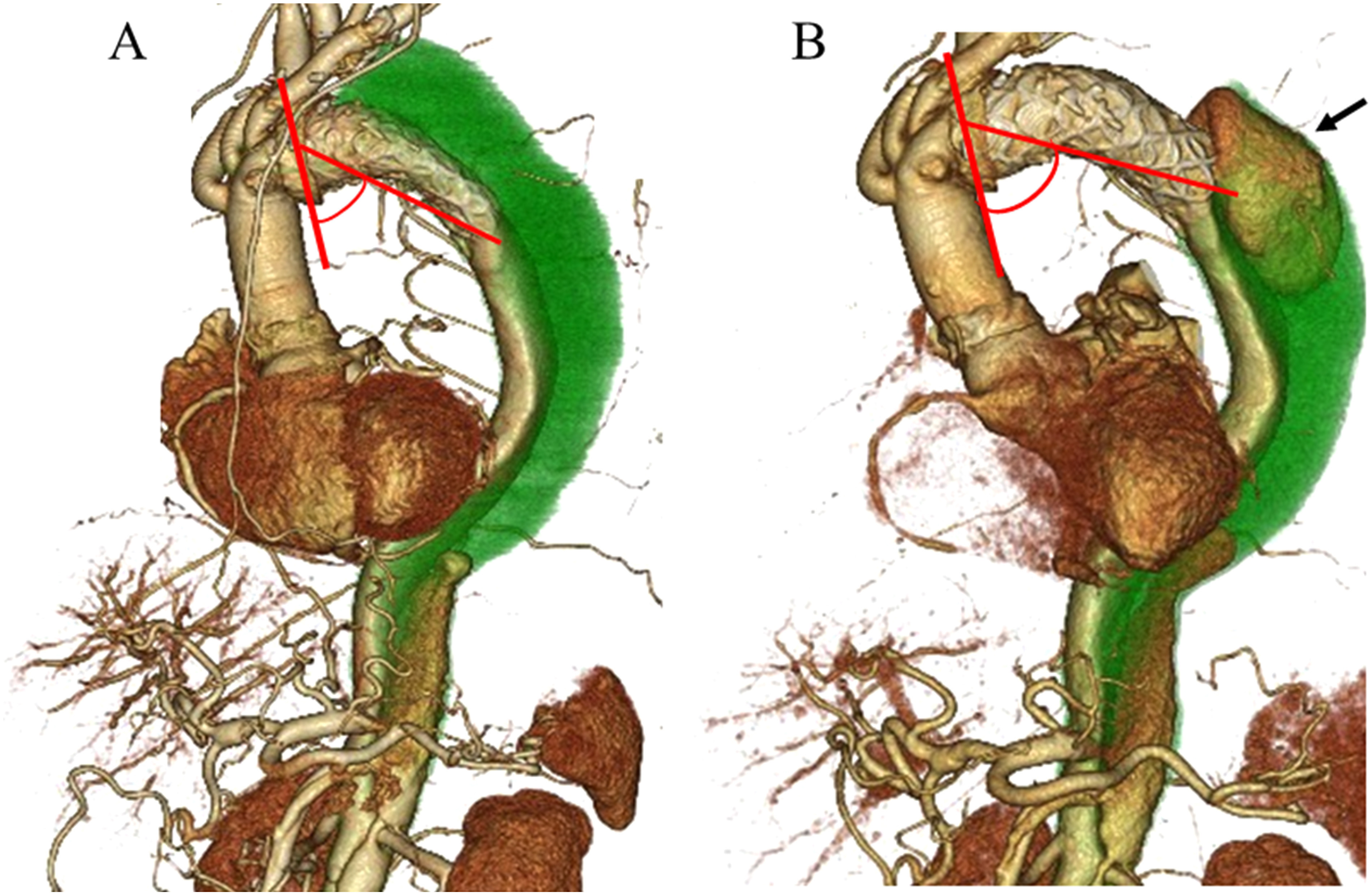
Fig. 2 Measurement of the angle between the line perpendicular to the distal anastomosis of the total arch replacement and the line from the center of the distal end of the frozen elephant trunk (FET) to the center of the proximal FET. (**A**) Postoperative 3D-computed tomography (CT) showing the angle of 52°. (**B**) Follow-up 3D-CT showing the angle of 60°. Distal stent graft-induced new entry is recognized at the distal edge of the FET (arrow).

The risk factors of dSINE are oversizing and springback.^10–12)^ The oversize rate was determined by dividing the aortic diameter and the size of the implanted FET for acute dissection as well as by dividing the true lumen diameter and the size of the implanted FET from the preoperative CT for chronic dissection. To evaluate the springback, the angle between the line perpendicular to the distal anastomosis of the TAR and the line from the center of the distal end of the FET to the center of the proximal FET was measured and compared postoperatively and during the follow-up period (**Fig. 2**). Aortic remodeling was defined as a decrease in the maximum aortic diameter greater than 5 mm.^13)^

### Statistical analysis

All values were expressed as mean±standard deviation; between-group comparisons between those who did and did not develop dSINE were performed using the Wilcoxon rank-sum test. The factors were divided into three periods as acute, subacute, and chronic during the comparison. These periods were defined by the 2020 Guideline on Diagnosis and Treatment of Aortic Aneurysm and Aortic Dissection.^9)^ Categorical variables were analyzed using the chi-squared and Fisher’s exact tests. Statistical analyses were conducted using the JMP software (SAS Institute Inc., Cary, NC, USA). Statistical significance was defined as P<0.05.

## Results

The mean patient age was 63.5±11.1 years (range, 40–85 years), and there were 54 men and 16 women. The patient characteristics are summarized in **Table 1**. In total, 59 (84.3%) and 11 (15.7%) patients had Stanford type A and type B aortic dissections, respectively. Postoperative results revealed SCI in six patients (8.6%), paraplegia in four (5.7%), and paraparesis in two (2.9%). The mean follow-up period was 24.7±13.6 months.

**Table 1:** Table 1 Clinical demographic patient characteristics

Variable	n=70
Age (years)	63.5±11.1
Sex	
Male	54 (77.1%)
Comorbidities	
Hypertension	58 (82.8%)
Diabetes mellitus	8 (11.4%)
Renal insufficiency	16 (22.9%)
Coronary artery disease	2 (2.9%)
Cerebrovascular disease	4 (5.7%)
COPD	3 (4.3%)
Stanford type A dissection	59 (84.3%)
Acute	40
Subacute	3
Chronic	16
Stanford type B dissection	11 (15.7%)
Acute	4
Subacute	3
Chronic	4

SD: standard deviation; COPD: chronic obstructive pulmonary disease

dSINE occurred in nine patients (12.9%) within a mean of 17.7±11.7 months postoperatively (range, 0.7–36.1 months). Among them, four (9.1%) and five (25%) patients who underwent dissection in the acute and chronic periods developed dSINE, respectively. Although the incidence tended to be higher in chronic dissection, the difference was not significant (P=0.08). None of the patients who underwent surgical intervention in the subacute phase developed dSINE. With respect to the incidence according to the type of dissection, six (10.2%) patients had type A dissection, whereas three had type B dissection (27.3%), with no significant difference (P=0.11) (**Table 2**).

**Table 2:** Table 2 Patient characteristics by occurrence of distal stent graft-induced new entry

	dSINE (n=9)	non-dSINE (n=61)	P value
Etiology of dissection			
Stanford type A	6 (10.2%)	53 (89.8%)	*0.11
Stanford type B	3 (27.3%)	8 (72.7%)	
Acute dissection	4 (9.1%)	40 (91.0%)	**0.08
Chronic dissection	5 (25.0%)	15 (75.0%)	
Subacute dissection	0 (0%)	6 (100%)	
Age	61.4±11.8	63.8±11.1	0.61
Men	6 (66.7%)	48 (78.7%)	0.42

*Stanford type A vs. type B dissection **Acute vs. chronic dissection dSINE: distal stent graft-induced new entry

The details of FET and postoperative angle changes in the distal edge of the FET were separately studied for acute and chronic dissection (**Table 3**). No difference was observed in FET size and length between the dSINE and non-SINE groups. The FET size was selected based on aortic diameter in acute dissection and true lumen diameter in chronic dissection. There were no significant between-group differences in the oversizing rates in both acute (dSINE group: 78.7% vs. non-dSINE group: 74.8%) and chronic (dSINE group: 105.3% vs. non-dSINE group: 105.2%) dissections. Although some patients with dSINE exhibited obvious springback, there was no significant difference in angle changes at the distal edge of the FET. In the dSINE group, all patients developed enlargement of the thoracic aortic diameter, including the FL, whereas thoracic aortic remodeling did not occur. On the contrary, remodeling of the thoracic aorta was only observed in 35 patients (57.3%) in the non-dSINE group (P=0.0013).

**Table 3:** Table 3 Interventional details and postoperative changes by occurrence of distal stent graft-induced new entry

	dSINE (n=9)	non-dSINE (n=61)	P value
FET size (mm)			
Acute dissection	26.5±1.9	25.6±2.5	0.10
Chronic dissection	26.6±3.3	27.0±4.1	0.85
FET length (mm)			
Acute dissection	82.5±15.0	80.2±20.8	0.70
Chronic dissection	102.0±16.4	100.0±21.7	0.96
Oversizing rate (%)			
Acute dissection*	78.7±8.5	74.8±8.5	0.48
Chronic dissection**	105.3±13.1	105.2±9.8	0.79
Distal edge angulation changes (degrees)			
Acute dissection	1.5±1.7	7.4±6.7	0.09
Chronic dissection	9.6±6.7	3.9±7.0	0.11

*Comparison of whole aortic diameter and FET size **Comparison of true lumen diameter and FET size dSINE: distal stent graft-induced new entry; FET: frozen elephant trunk

### Reintervention after initial development of dSINE

**Figure 3:**
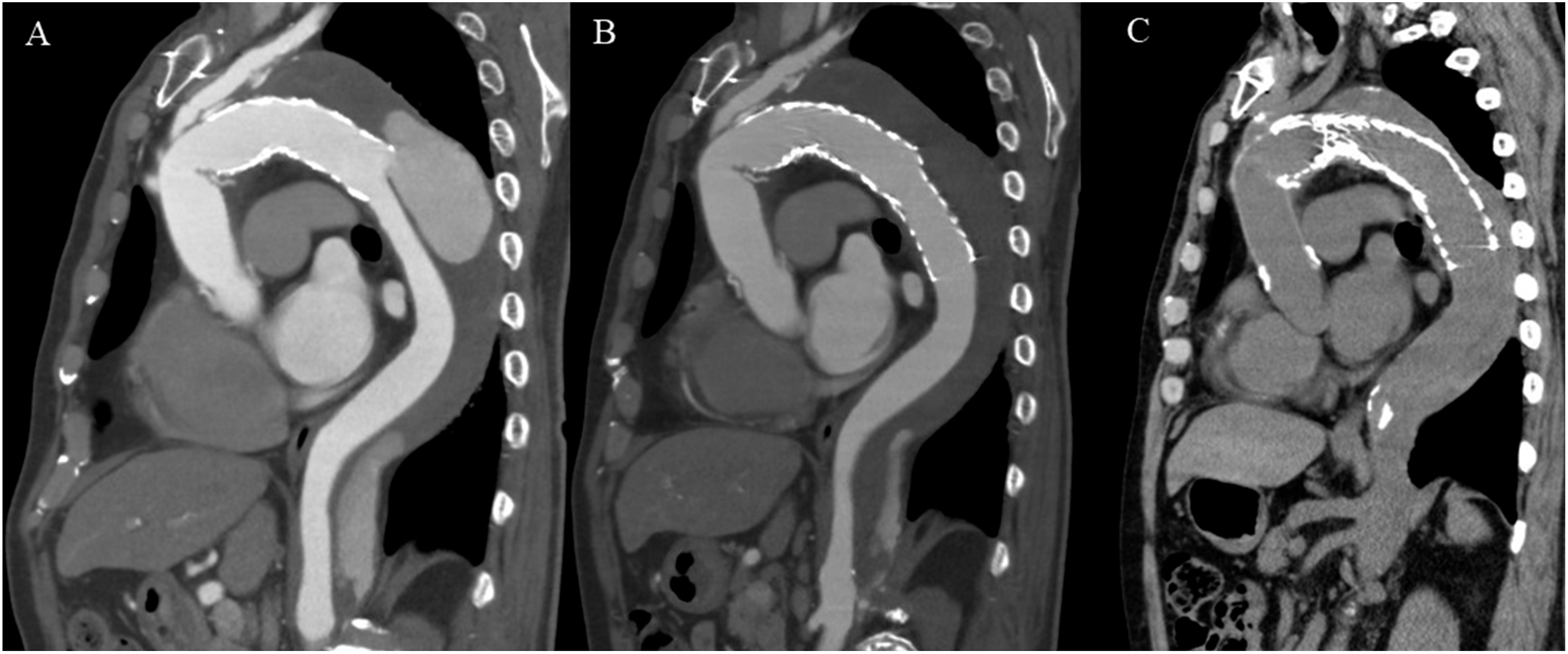
Fig. 3 (**A**) Distal stent graft-induced new entry is observed 1 year after surgery. (**B**) After thoracic endovascular aortic repair (TEVAR). (**C**) Regression of the false lumen of the thoracic aorta is observed 2 years after TEVAR.

All patients in the dSINE were treated with TEVAR due to thoracic aortic enlargement (**Fig. 3**). No TEVAR-related complications (e.g., SCI) occurred, and thrombosis of the thoracic FL was observed in all patients.

## Discussion

The advantages of FET are that it can cover the entry of the distal arch and descending aorta, and the true lumen diameter can be secured with malperfusion from the median sternotomy approach. When the FL is patent after the initial operation, the 10-year rate of additional surgical procedure due to descending aortic expansion ranges from 16% to 26%, and the surgery includes left thoracotomy, re-sternotomy, or TEVAR.^14,15)^ Many reports have demonstrated the feasibility of the FET technique for acute aortic dissection to minimize the incidence of FL patency.^3,5,6,16–20)^

Another advantage is that FET saves on procedure-related times, including operation and cardiopulmonary bypass.^18)^ This may be because the SG is inserted into the distal true lumen, reinforcing the distal anastomosis. However, FET also has the disadvantages of SCI and dSINE. Neurological complications, particularly SCI, have been reported in approximately 5% of the patients.^21,22)^

Compared with a stent length of 10 cm, a stent length of ≥15 cm is associated with a higher SCI rate. The rate is also higher when the coverage is Th8 or beyond.^21)^ In this study, although we set the distal FET implantation position to not exceed Th8, SCI still occurred in 8.6% of the patients. However, there was a possible cause other than FET in three of paraplegia cases. In one case, paraplegia was present preoperatively and was caused by malperfusion of the intercostal artery due to aortic dissection, which did not improve postoperatively. Another case may have been caused by cardiac arrest from hypotension during postoperative dialysis. The third case was caused by malperfusion of the intercostal artery owing to intraoperative FL perfusion. In addition to the distal position of the SG, intraoperative and postoperative blood pressure, atheromatous emboli of the spinal cord artery, and duration of circulatory arrest also increase the risk of neurologic complications.^17,21,22)^

Recent reports indicated that dSINE occurs in 15.8%–18% of patients following FET for aortic dissection.^6,7,12)^ Furthermore, the incidence has been reported to increase yearly after surgery.^12,23)^ Meanwhile, the incidence of dSINE with conventional TEVAR for aortic dissection is approximately 1.08%–34.7%.^11)^ Various risk factors of dSINE have been reported, including TEVAR for chronic aortic dissection, oversizing, springback force, expansion force of the SG itself, aortic remodeling mismatch, and SG length less than 145 mm.^10–12,23,24)^ The dissected aorta morphology changes over time, the intimal flap becomes thicker and less mobile.^25)^ Thus, after TEVAR and FET for chronic dissection, the dissected intimal flap cannot reapproach the aortic outer wall due to the changes in aortic morphology. This may lead to dSINE at the distal part of the device over time.^10)^ In this study, dSINE was more common in chronic dissection than in acute dissection (25% vs. 9%), but the difference was not significant.

In contrast to other reports, we found no difference in the oversizing rate for FET size selection, which can be up to 90% of the whole aortic diameter in the acute phase^6,7,17)^ and 10–20% of the true lumen diameter in the chronic phase.^19,26)^ The oversizing rates were 78.7% and 74.8% in the dSINE and non-SINE groups for acute dissection and 105.3% and 105.2% in the dSINE and non-SINE groups for chronic dissection. Selecting a smaller FET will reduce the risk of dSINE but may not allow distal type Ib endoleak or entry closure. Further, it is difficult to determine the pre-dissection aortic diameter, and dissection itself causes the aortic diameter to increase by approximately 8%.^17)^ Another report has demonstrated a +23% increase in the mid-descending thoracic aortic diameter in patients with acute type B aortic dissection.^27)^ Yamauchi et al. proposed a parameter that calculates the pre-dissection aortic diameter based on the circumference length of the true lumen and whole aorta at the time of dissection.^28)^ This method could possibly optimize the selection of FET size.

Springback is also the most relevant risk factor for dSINE. Because Frozenix has a strong springback force that makes it try to straighten itself, placing it in a shallow position may put more force on the FL wall and increase the risk of dSINE.^7)^ In this study, although some patients with dSINE exhibited obvious springback, no significant difference was observed in angle changes at the distal edge of FET. There is a report that experimentally verified the ex vivo expansion force of the SG itself in Thoraflex (Vascutek Ltd., Inchinnan, United Kingdom) and E-vita (Jotec Inc., Hechingen, Germany). It was reported that the distal edge of the Thoraflex was stiffer than that of E-vita.^12)^ The expansion force of Frozenix also needs to be compared and investigated in a similar experiment.

The aortic remodeling mismatch caused by FL regression of the SG-covered aorta and FL non-regression of the non-SG-covered aorta is also thought to be a factor affecting the development of dSINE.^12,24)^ The stent-flap angle is reduced by the aortic remodeling mismatch, and the retrograde blood flow in the FL causes mechanical stress at the distal edge of the FET, resulting in dSINE. However, there were no causes of aortic remodeling mismatch in this study.

### Study limitations

This study has limitations, including the retrospective study design, short follow-up duration (mean, 2 years), and limited number of patients. Larger studies with longer observation periods are warranted to establish the risk of dSINE after FET for aortic dissection.

## Conclusion

The presence of dSINE requires lifelong monitoring as it increases the risk of aortic diameter enlargement. Although several risk factors have been identified in previous studies, this study did not identify any independent clinical or individual factor predicting the development of dSINE. Selecting a smaller FET size for the sake of avoiding dSINE is not recommended, as it will not result in the expansion of the true lumen and aortic remodeling. Additional TEVAR for dSINE provides good results, with no complications, and achieves FL thrombosis in the thoracic aorta.

## Additional Note

The outline of this report was presented at the 61st Annual Meeting of the Japanese College of Angiology on October 13, 2020, in Sendai, and was recommended by the chairperson.
